# Boxes of rain: A systematic review on the classes and frameworks of ecosystem disservices

**DOI:** 10.1007/s13280-025-02157-1

**Published:** 2025-03-13

**Authors:** Carl Cyrus Anderson, Andreas Metzemacher, Blal Adem Esmail

**Affiliations:** 1 Institute of Environmental Planning, Leibniz Universität Hannover, Herrenhäuser Str. 2, 30419 Hannover, Germany; 2 Institute of Geography, Ruhr University Bochum, Universitätsstr. 150, 44805 Bochum, Germany; 3 Kreisstadt Bergheim, Urban Planning Department, Bethlehemer Str. 9-11, 50126 Bergheim, Germany; 4 Center for Global Mountain Safeguard Research (GLOMOS), Eurac Research, Drususallee/Viale Druso 1, 39100 Bozen, Bolzano Italy

**Keywords:** Classification, Ecosystem disservices, Ecosystem services, Framework, Planning and management, Urban green infrastructure

## Abstract

Despite the numerous benefits we derive from ecosystems as ecosystem services (ES), negative impacts on human health and well-being also occur as ecosystem disservices (ED). To study ED, researchers box them into classes and create conceptual frameworks to support their identification, assessment, and communication. It is unclear what classes and frameworks now exist for ED, their relative uptake in the literature, and their potential application across socio-ecological contexts. We conduct a systematic literature review of ED classification systems and frameworks to take stock and synthesize this growing but scattered body of research. We find strong uptake of several influential articles but persisting inconsistency of classifications and oversimplification of ED processes within frameworks. Aggregating existing classifications and frameworks, we draw on lessons learned to propose the Composite Ecosystem Disservices (CED) framework. We call for increased ED research, greater comparability and replicability allowing for transdisciplinarity, and conceptual underpinnings that recognize social-ecological interconnections.

**Supplementary Information:**

The online version contains supplementary material available at 10.1007/s13280-025-02157-1.

## Introduction

Ecosystem services (ES) are the direct or indirect benefits that humans derive from ecosystems for survival and quality of life^1^ (MEA 2005; Harrington et al. 2010; TEEB 2012; Costanza et al. 2017). Green spaces perform numerous functions such as regulating air quality, stormwater, and local temperatures, reducing noise, providing recreational opportunities, and enhancing social cohesion (Gómez-Baggethun and Barton 2013; Veerkamp et al. 2021). This ultimately can improve people’s physical and mental health as well as overall well-being. However, humans can also be negatively impacted by the functions, attributes, and processes of ecosystems. These impacts (or trade-offs) can be referred to as ecosystem disservices (ED) (Döhren and Haase 2015).

ED are defined as ‘ecosystem-generated functions, processes, and attributes that result in perceived or actual negative impacts on human well-being’ (Shackleton et al. 2016). Subjective values and perceptions, as well as individual experiences, are central to understanding both ES and ED. Perceptions can vary with demographic factors like age or education (Fernandes et al. 2019) and divergent demands on the respective element or space (Lyytimäki et al. 2008; Baumeister et al. 2022). This is exemplified in urban contexts with high density and diversity of people in contact with green spaces or features (i.e., ‘urban green infrastructure’ or UGI) (Pauleit et al. 2019). For example, trees in urban green spaces illustrate the potential of both perceived and actual ES and ED originating from the same ecosystem (element). While some people perceive trees positively, e.g., as an aesthetically pleasing source of shade and thermal comfort, or as a way to connect with nature, others may suffer allergic reactions or incur costs due to falling leaves or limbs and damaged infrastructure (Baumeister et al. 2022; Drew-Smythe et al. 2023). Beyond the individual, context is also crucial, as the same trees potentially valued for shade during the day may cause fear or danger at night due to an increased risk of criminality in dark or hidden spaces (Drew-Smythe et al. 2023).

Despite the importance of such disservices and the potential for planning and management to address them (at least partially), there is still a wide disparity in research between ES and ED. This stems from the greater policy support and endorsement that the ES concept has received through initiatives like the Millennium Ecosystem Assessment (MEA) (MEA 2005), The Economics of Ecosystems and Biodiversity (TEEB) (TEEB 2012), and the Intergovernmental Science-Policy Platform on Biodiversity and Ecosystem Services (IPBES) (e.g., IPBES 2022). While there has been an increase in published articles on ED (Döhren and Haase 2015), the number is still very limited compared to studies focusing on ES. In a literature search conducted in March 2019 in the core collection of the web of knowledge, Blanco et al. (2019) found 27,441 articles on ES and 194 on ED.

Beyond the relatively small number of studies examining ED, Guo et al. (2022) found that terminological and conceptual ambiguity surrounding ED complicates interdisciplinary collaboration, as scholars and stakeholders may interpret and prioritize ED differently. This lack of consensus has been noted as a key barrier to cohesive research and policy integration on ED (Vaz et al. 2017; Guo et al. 2022). In contrast, the ES concept, despite targeted criticism of an overly anthropocentric and commodity-oriented approach (Schröter et al. 2014), has been successful at promoting an inter- and transdisciplinary understanding of nature’s benefits while also providing a crucial boundary concept to planning and policy (Abson et al. 2014; Adem Esmail and Geneletti 2017). This success suggests that conceptual clarity and policy backing can greatly enhance a framework’s interdisciplinary and practical application. Although the ES field contains diverse classifications and frameworks, such as the Common International Classification of Ecosystem Services CICES, this has not hindered its coherence or effectiveness, indicating that multiple frameworks can coexist without diminishing a concept's impact. Given these observations, building a general consensus on the ED concept is one important step toward evidence-based assessment and management (Saunders 2020). While consensus alone may not address all challenges facing ED research, establishing clearer definitions and classifications could facilitate interdisciplinary collaboration and increase the concept’s utility in policy contexts (Saunders 2020).

According to Blanco et al. (2019), although ED is still most often an afterthought within ES studies, conceptual frameworks of ED have advanced along with empirical research on the topic (Lyytimäki and Sipilä 2009; Döhren and Haase 2015; Shackleton et al. 2016). Nonetheless, ED conceptual frameworks and classifications have not enjoyed much benefit from standardization and replicability (Campagne et al. 2018; Blanco et al. 2019; Guo et al. 2022) and the concepts of ES and ED have mostly been integrated only by adding ‘ED’ as a single extra class of generic trade-offs within ES-focused studies. However, several papers have attempted to develop frameworks and classifications that more deeply integrate the two approaches. For example, Lyytimäki (2017) developed a framework where ES and ED arise equally from ecosystem functions, with several factors such as context, scale, and urban forest heterogeneity influencing the magnitude of ED. Vaz et al. (2017) proposed a comprehensive typology for ED based on a systematic review, providing a framework for integrating ES and ED for human well-being in relation to ecosystem functions. Later, Mao and Cui (2021) presented ED as an indirect product of ecosystems. The authors integrated time, space, and stakeholders into their framework, but without specifying how ES and ED are generated. Escobedo et al. (2011) also proposed a classification and framework for ED, using the example of urban forests, with a focus on the balance between ED and air pollution mitigation potential. Their classification divides ED into financial, social, and environmental groups, while their simple framework shows how the characteristics and management of urban forest can provide pollution mitigation, but also ED. Ceauşu et al. (2019) focus on human–wildlife interactions and management and present the ‘SEEDS’ framework (social-ecological framework for ecosystem disservices and services) which conceptualizes governance, wildlife areas, and ED recipients as creating links between possible interactions and outcomes.

These articles demonstrate the increased research interest on ED, not only practically but also conceptually. However, findings in relation to different frameworks and classifications applied in academic articles are mostly given only brief mention. It is unclear how many ED classifications and frameworks now exist in the literature, to what degree they have been taken up, as well as their similarities, differences, and relative pros and cons. Additionally, existing classifications and frameworks are often derived from specific green features such as urban trees (Escobedo et al. 2011; Roy et al. 2012) or specific geographic extents (Lyytimäki et al. 2008). This creates a knowledge gap regarding how applicable existing ED conceptualizations are across contexts, as well as their degree of corresponding convergence or divergence. A recent review of ES and ED by Nápoles-Vértiz and Caro-Borrero (2024) includes a discussion of conceptual approaches, but the primary contribution is the synthesis of three challenges in ES and ED research: (1) lack of understanding and standardization of classifications; (2) identification of the co-production by humans and ecosystems; and (3) interactions across spatial and temporal scales. In this paper, we aim to address the first challenge and expand it to include conceptual frameworks.

We systematically document existing ED classification systems and conceptual frameworks, as well as aggregate them to create a widely applicable classification and framework to support awareness, understanding, assessment, and management of ED. To accomplish this, we examine the individual ‘boxes’ (e.g., of rain)^2^ that represent single concepts and classes used in the construction of frameworks and classification systems. We conduct a targeted systematic literature review of past ED research, also including studies that do not use the term ED explicitly. Our approach is guided by two main research questions that correspond to the goals of understanding, classifying, and conceptualizing ED:

1. How have ED been classified in past research and what has been the relative uptake of these classifications?
2. How have ED been conceptualized within frameworks in past research and what has been the relative uptake of these frameworks?

We compile lessons learned from the review in our novel Composite Ecosystem Disservice (CED) framework to a) synthesize the knowledge gained during the review and b) orient the findings toward the management and mitigation of ED.

## Ecosystem disservices bibliometric overview

Before addressing our research questions, we first provide here a bibliometric overview of influential works, thematic trends, and geographic distribution of the ED research field, building on existing reviews. We use 535 articles returned by the key word sequence ‘ecosystem NEAR/2 disservice* OR ecosystem NEAR/2 dis-service* OR ecosystem NEAR/2 (dis)service*’ in Web of Science (on October 26, 2024, with no date limitation). Bibliometric analysis^3^ shows that Lyytimäki and Sipilä (2009) is the most highly cited article, followed by Döhren and Haase (2015) and Shackleton et al. (2016). ‘Ecosystem services’ is the most common keyword, which frequently co-occurs with ‘ecosystem disservice’ (and disservices). Other keywords include biodiversity, green infrastructure, climate change, and urban planning. The three most popular journals are Ecosystem Services, Urban Forestry and Urban Greening, and Land (see Figure S1 in supplementary material).

**Fig. 1:**
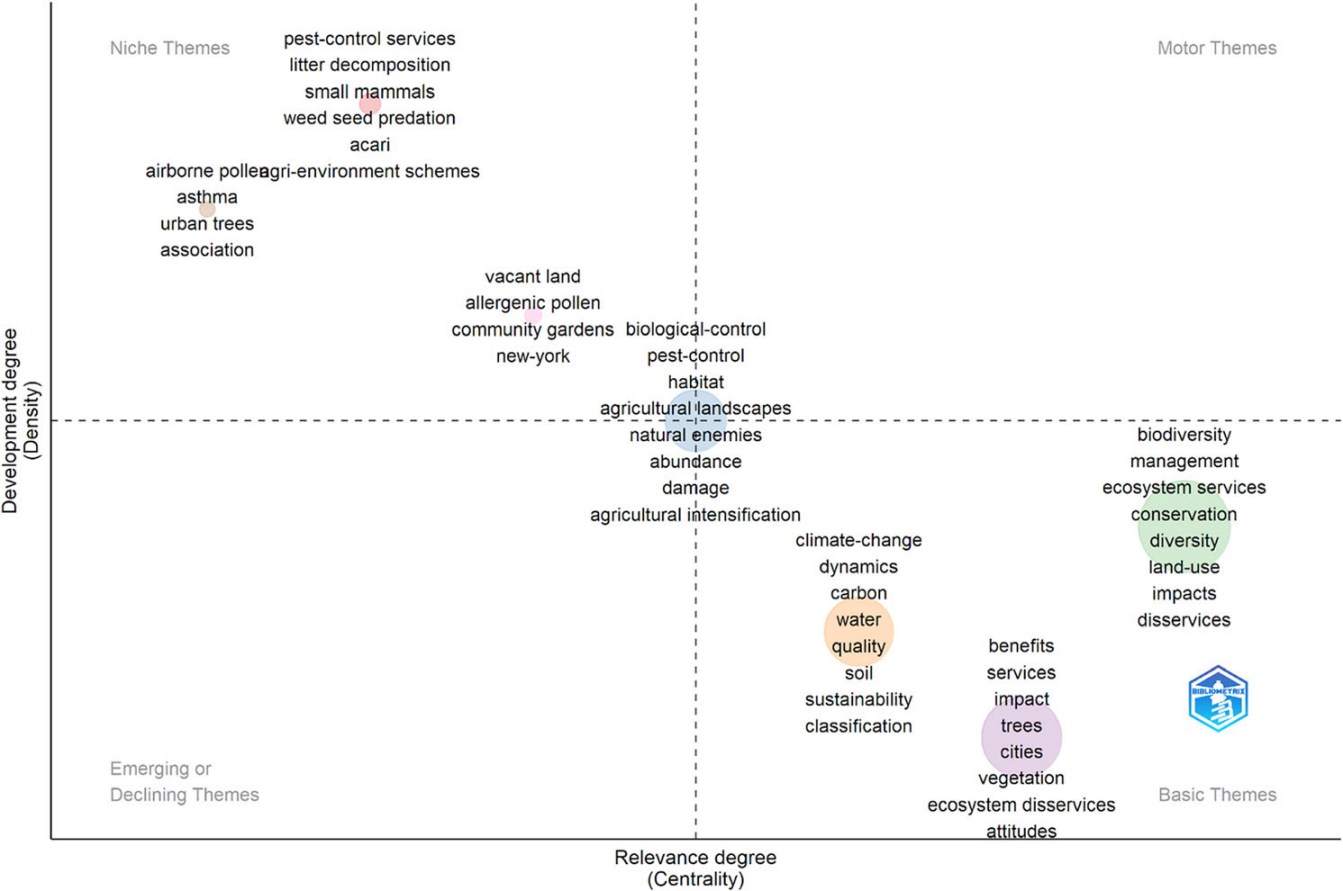
Thematic mapping plot of KeyWords Plus generated using Bibliometrix (Aria et al. 2022) within the 535 articles in the larger ED dataset

The vast majority of author affiliations are from USA-based institutions (n = 352), followed by the UK (157), Germany (138), China (110), and France (89). Thematic mapping of ‘KeyWords Plus’ (derived from the titles of cited references) shows clusters within higher values of density and lower values of centrality (i.e., top-left quadrant—*niche themes*) related mostly to pests and allergies. These are strongly developed topics but do not cut across the literature (Fig. 1). ‘Basic themes’ (high centrality and low density) are significant for the domain and cross-cutting to its different areas (Aria et al. 2022). Three clusters emerge: from left to right in the bottom right quadrant—1) climate change and environmental processes; (2) urban services and disservices including more articles studying tree impacts and public attitudes; and 3) services and disservices broadly with focus on biodiversity and conservation.

## Methods

### Literature search

To answer our research questions on ED classifications and frameworks, we started with the set of 535 ED articles based on the same sequence used for the bibliometric review presented above (‘ecosystem NEAR/2 disservice* OR ecosystem NEAR/2 dis-service* OR ecosystem NEAR/2 (dis)service*’ input in Web of Science on October 26, 2024 with no date limitation). We then followed PRISMA guidelines (Page et al. 2021) to systematically exclude articles based first on title, then abstract, then full text so that only articles using an existing, adapted, or new classification and/or framework of ED were included in the final set. We define ‘classification’ as a system to group ED (into boxes). Articles that only describe a list of individual ED but do not group them were excluded (e.g., Drew-Smythe et al. 2023). We excluded the many articles that assess ES and only classify ED generally as a separate ‘catch-all’ theme. We define ‘framework’ broadly as a graphical representation of elements, processes, and their relationships. We exclude articles that only include ED as an explanatory factor toward an outcome in a path diagram (e.g., ED effects on behavioral intention (Wu et al. 2022)) since frameworks must include ED as a key element or focus with some systemic and interconnected elements (e.g., origin, impacts, ES, etc.).

Of the included articles that use a new or adapted classification or framework, we distinguish between those suited for application across contexts and those that are context-specific. The distinction follows the simple question ‘Could this classification or framework be applied in many different places (social-ecological systems)?’. For example, Barnes et al. (2020) classify ED of residential yards in Minnesota (context-specific), compared to classifying the ED of a broader concept such as ‘urban forests’ (e.g., Escobedo et al. 2011) or street trees (e.g., Döhren and Haase 2019) (cross-context).

After applying the above exclusion criteria and conducting stepwise title and abstract exclusion, 44 articles remained. We then used ad hoc Web of Science and Google Scholar searches combined with snowball sampling to include further articles by checking citations in the 44 relevant articles. The searches in Google Scholar are based on sets of key words describing ED not only using explicitly ‘ecosystem’ and ‘disservices,’ but including synonyms (Table 1). These keywords originate from the initial article screening, author knowledge, as well as Lyytimäki and Sipilä (2009) in their discussion of ‘defining disservices.’

**Table 1:** Search terms combined for ad hoc supplement literature search beyond the use of explicitly ‘ecosystem’ and ‘disservice.’ All combinations of group 1, 2, and 3 terms were tested with ‘AND’ operators

1—Ecosystem	2—Disservices	3—Objects
Ecosystem	Trade-off	Effect
Ecologic	Disbenefit	Impact
Nature	Nuisance	Consequence
Green	Conflict	
	Disturbance	
	Disconnection	
	Fear	
	Unpleasant	
	Avoid	
	Harmful	
	Unwanted	
	Undesired	
	Negative	

**Fig. 2:**
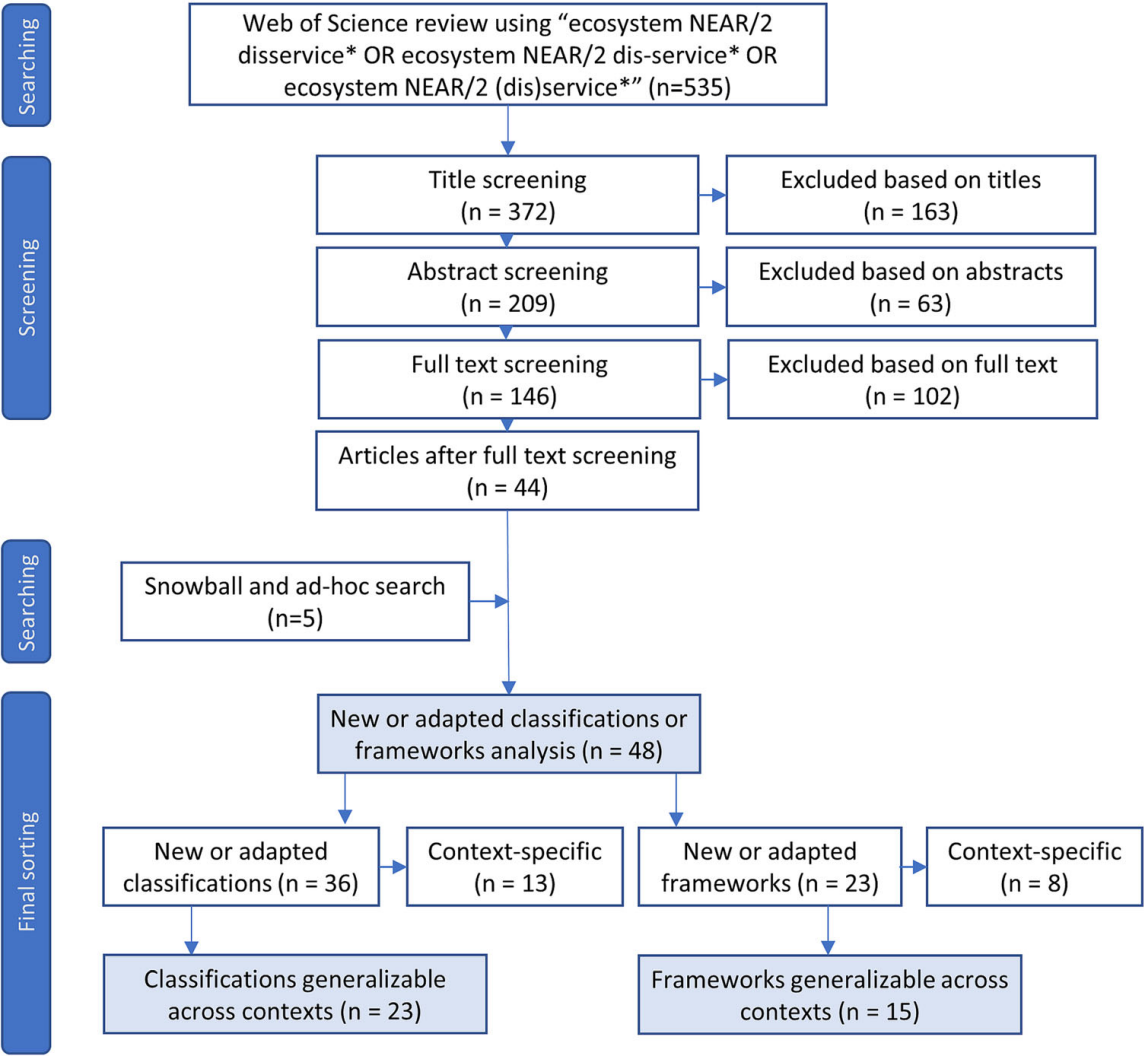
Systematic review process with included/excluded articles at each step. Light blue boxes represent the final data sets for analyses. 11 articles contain both a new or adapted framework and classification system. PRISMA diagram from Page et al. (2021)

This resulted in 5 additional articles after screening, bringing the total to 48 (see full list in Text S1); 36 that include a new or adapted classification and 17 with a new or adapted framework. Seven articles include both a new classification and framework. Of the 36 classification articles and 23 framework articles, we focus our analysis on 23 and 15 articles, respectively, that include classifications or frameworks generalizable across contexts (Fig. 2).

### Data analysis

The 48 papers were inductively coded and analyzed using the knowledge and reference management software Citavi (v6). Text and figures referring to classifications and frameworks were assigned to corresponding codes (i.e., ‘categories’ in Citavi). Further sub-codes were used for individual unique classes of ED, and articles including these classes were assigned to the codes accordingly. Classification results are presented as tables describing the authors’ given purpose for the classification, the unique classes, and any defining features. We also list the number of citations that the article describing the classification has accrued, as an indication of its relative uptake in research. Lastly, we show a comparison of classification systems based on aggregate classes—ecological, economic, health and safety, and social and cultural (Döhren and Haase 2015; Roman et al. 2021; Lategan et al. 2022) to identify convergence and divergence across articles.

Text describing elements and connections within frameworks was also coded. All frameworks were assessed by the author team regarding their key components, unique elements, and origin (i.e., if they were adapted from or based on other ES or ED frameworks). As a final step, we aggregated findings across articles through the creation of an overarching ED framework—the Composite Ecosystem Disservice (CED) framework. The CED framework thereby represents conceptual consensus on ED while including the unique contributions of classification and framework articles most suited for the cross-context management and mitigation of ED.

## Results

### Classifications of ecosystem disservices

We found 36 articles that describe and/or apply a new or adapted ED classification. Of these, 13 articles describe context-specific classifications including the ED of the auroras (Broome et al. 2024), gentrifying neighborhoods (Rodgman et al. 2024), and specific invasive species (Milanović et al. 2020), which are described in more detail in supplementary material (Table S1).

In this section, we focus on 23 articles we identified that include a new or adapted classification that is generalizable across contexts. Within these, 12 articles explicitly propose an ED classification and 11 articles apply a classification (without explicitly encouraging its uptake) (Table 2).

**Table 2:** List of articles that (a) include a new or adapted classification that is replicable across contexts and explicitly propose an ED classification or (b) apply a classification (but do not explicitly encourage its uptake)

a)				
Article	Citations^1^	Classification purpose/topic	Classes	Defining features
Opoku et al. (2024)	2	Urban tree ES and ED through case study in Kumasi, Ghana	Aesthetic and environmental issues; safety and security issues; health issues; mobility and infrastructure issues	Adapted from 10 different sources, including Escobedo et al. (2011), Lyytimäki (2014); Drew-Smythe et al. (2023)
Uchida et al. (2024)	4	Human–wildlife interaction	Economy, health and safety, esthetics and culture	Focus on fauna and their tolerance to human interactions as the origin of ED
Guo et al. (2022)*	17	Indicator system for urban ED research and assessment	Agriculture, urban, urban forest, urban birds, plant invasions, environment	Based on ‘ecological context’
Lliso et al. (2022)	33	Describe ‘nature’s contributions to people’ (NCP) theory	(Dis)values as intrinsic, instrumental, relational	Aims to shift away from ES concept
Soga and Gaston (2022)*	26	Negative direct human–nature interactions	Tactile, visual, auditory, olfactory; also described in terms of more/less frequency, intensity, and consistency	Emphasis on human senses and how animals, plants, and ecosystems create pathways to negative interactions
Ceauşu et al. (2019)	111	ES and ED in human–wildlife interactions	Damage to livestock, crops, property; loss of human life, health; competition for resources + local, regional, national scale	Part of a social-ecological framework systematic, qualitative analysis of human–wildlife systems. More suited for rural settings
Cariñanos et al. (2017)	63	Urban trees	Environmental/ecological, health hazards, economic costs, social hazards	Classes adapted from Escobedo et al. 2011; Roy et al. 2012; Gómez-Baggethun and Barton 2013, Döhren and Haase 2015
Vaz et al. (2017)*	253	Comprehensive ED typology using plant invasion as an example	Health, material, security and safety, cultural and aesthetic, leisure and recreation	Classes are tied to potential negative impacts on well-being, albeit 'material' relates to damaging built infrastructure
Shackleton et al. (2016)	390	Ecosystem origin and impact on human well-being	Economy, health, cultural; subdivided into ‘biotic’ and ‘abiotic’	Natural hazards feature strongly as examples of abiotic, since they ‘are a result of changes in ecological and/or biological processes of an ecosystem’
Döhren and Haase (2015)*	511	Impact of ecosystem processes on human quality of life	EEcological impact, economic impact, health impact	ED as impacts clustered ‘along thematic fields’
Escobedo et al. (2011)*	1425	Costs and ED of urban forests	Financial (land, labor, and capital), social nuisances, environmental	Expands understanding more broadly to associated costs
Lyytimäki et al. (2008)	415	Potential negative impacts of ecosystems to lifestyle	Aesthetic issues, safety issues, security and health issues, economic issues, mobility issues	Modified from Petersen et al. (2005)

b)				
Article	Citations^1^	Purpose/topic	Classes	Defining features
Andrade et al. (2024)*	2	Plant species as urban green infrastructure	Native vs. exotic as well as species invasiveness, species toxicity, branches collapse, pests and diseases, allergenic	ED classes used to organize findings of a review article
Zhang and MacKenzie (2024)*	7	Green infrastructure ES and ED	Mental health, economic, ecological	ED foci in reviewed academic literature for green infrastructure
Lategan et al. (2022)	12	Globally applicable to apply in Sub-Saharan Africa context since lack of past specific research	Environmental/ecological; economic/financial; health (physical, mental, safety); cultural (aesthetic and cultural); *subdivided into ‘biotic’ and ‘abiotic’*	Based on Lyytimäki and Sipilä (2009); Cilliers et al. (2013); Lyytimäki (2014); Döhren and Haase (2015); Lategan and Cilliers (2016); Shackleton et al. (2016); Davoren and Shackleton (2021)
Pistón et al. (2022)	15	ES and ED of street trees	Material, costs, aesthetic, safety	Classes to summarize data on perceptions in relation to social equity
Stroud et al. (2022)*	26	Present ED from 'urban plant ecology literature'	Health, material, cultural and aesthetic, ecological, security and safety, leisure and recreation, generic	ED classes only presented as ‘themes’ within a graph of review results
Roman et al. (2021)	238	Urban tree disservices across contexts	Infrastructure conflicts, human health and safety, cultural aesthetic, and social issues' environmental and energy issues	Adapted from Lyytimäki (2017) and Vaz et al. (2017)
Semeraro et al. (2021)	187	Urban green spaces	Photosynthesis; vegetation biomass growth; flow of floral gametes such as pollen; plants aging; dense development of the plants; decomposition and biomass root fixation; habitat provision for animal species; water supply; soil erosion	Classes defined by ecosystem functions. Adapted from Gómez-Baggethun and Barton (2013); Döhren and Haase (2015)
Potgieter et al. (2017)	126	Global urban invasive plant species	Cultural and aesthetic; economic problems; environmental problems; health; leisure and recreation; material; security and safety	Based on Roy et al. (2012) and Vaz et al. (2017)
Lyytimäki (2014)	120	ED appearing in a Finnish newspaper	Weather-related events, fears and risks, aesthetic issues, inhibition of activities, ecosystem functions causing harm	Further subdivided by ‘ecosystem function or property’
Gómez-Baggethun and Barton (2013)	2227	Provide examples of ED in urban areas	Air quality problems, view blockage, allergies, accidents, fear and stress, damages on infrastructure, habitat competition with humans	ED align with ecosystem functions—Photosynthesis, tree growth through biomass fixation, movement of floral gametes, aging of vegetation, dense vegetation development, biomass fixation in roots, decomposition; habitat provision for animal species
Roy et al. (2012)*	1276	Urban tree disservices across contexts	Social problems/hazards, economic problems/hazards, health problems/hazards, visual and aesthetic problems/hazards, environmental problems/hazards, costs and expenditures	Adapted from Groot et al. (2010)

^**1**^Citation numbers using Google Scholar—10 December 2024

*Review article

The first identified ED classification system was presented Lyytimäki et al. (2008), in which aesthetic, safety, security and health, economic, and mobility issues form boxes of ED. This influential contribution highlights the conflict between efforts at increasing biodiversity and social responses while sparking a tradition of urban ED research, in this case in the Northern European context. Escobedo et al. (2011) is noteworthy given its very high impact on subsequent literature as well as its explicit aim to ‘integrate the concepts of ecosystem services and disservices when assessing the efficacy of using urban forests for mitigating pollution.’ They generally refer to disservices as costs, which were further divided into financial, social nuisance, and environmental costs. Vogt et al. (2015) also describe costs of maintaining and managing urban forests—direct, infrastructure inference, externality-related, and opportunity costs. The direct costs include maintenance of green spaces, and the infrastructure interference costs refer to the fixing of urban infrastructure, such as sidewalks, because of damage by vegetation. The externality-related costs include allergies caused by pollen or the cleaning of green waste (i.e., outcomes that are not accounted for) while the opportunity costs include those associated with alternative, hypothetical land uses such as planting trees or building infrastructure like parking lots (Vogt et al. 2015). To show the costs of damaged infrastructure, Vaz et al. (2017) instead use the category of ‘material,’ later adapted by Roman et al. (2021) as ‘infrastructure conflict.’

The articles presenting or describing replicable classification systems use the term ‘ecosystem disservice’ with the exception of Soga and Gaston (2022) and Lliso et al. (2022). Soga and Gaston (2022) use ‘negative direct human–nature interactions’ to differentiate their conceptual approach from ED given a focus on harm done to humans through sensory pathways (e.g., animal stings and bites) and not vice versa. Classes are derived from ED origin—animals, plants, or ecosystems (e.g., the ‘visual’ sensory pathway includes scary animal encounters, seeing harm to plants, and seeing others suffer injury in natural contexts). Lliso et al. (2022) is based on the values used in ‘the IPBES approach’ as presented in Pascual et al. (2017), in which an alternative conceptualization to ecosystem services as ‘nature’s contributions to people’ (NCP) and the corresponding ‘disvalues’ are presented. Pascual et al. (2017) have over 1800 citations (December 2024), but we only found one article that takes up the NCP concept in the context of ED. Nicolás-Ruiz et al. (2024) describe ‘detrimental NCP’ classes in the context of dry rivers with a case study in southeastern Spain.

**Fig. 3:**
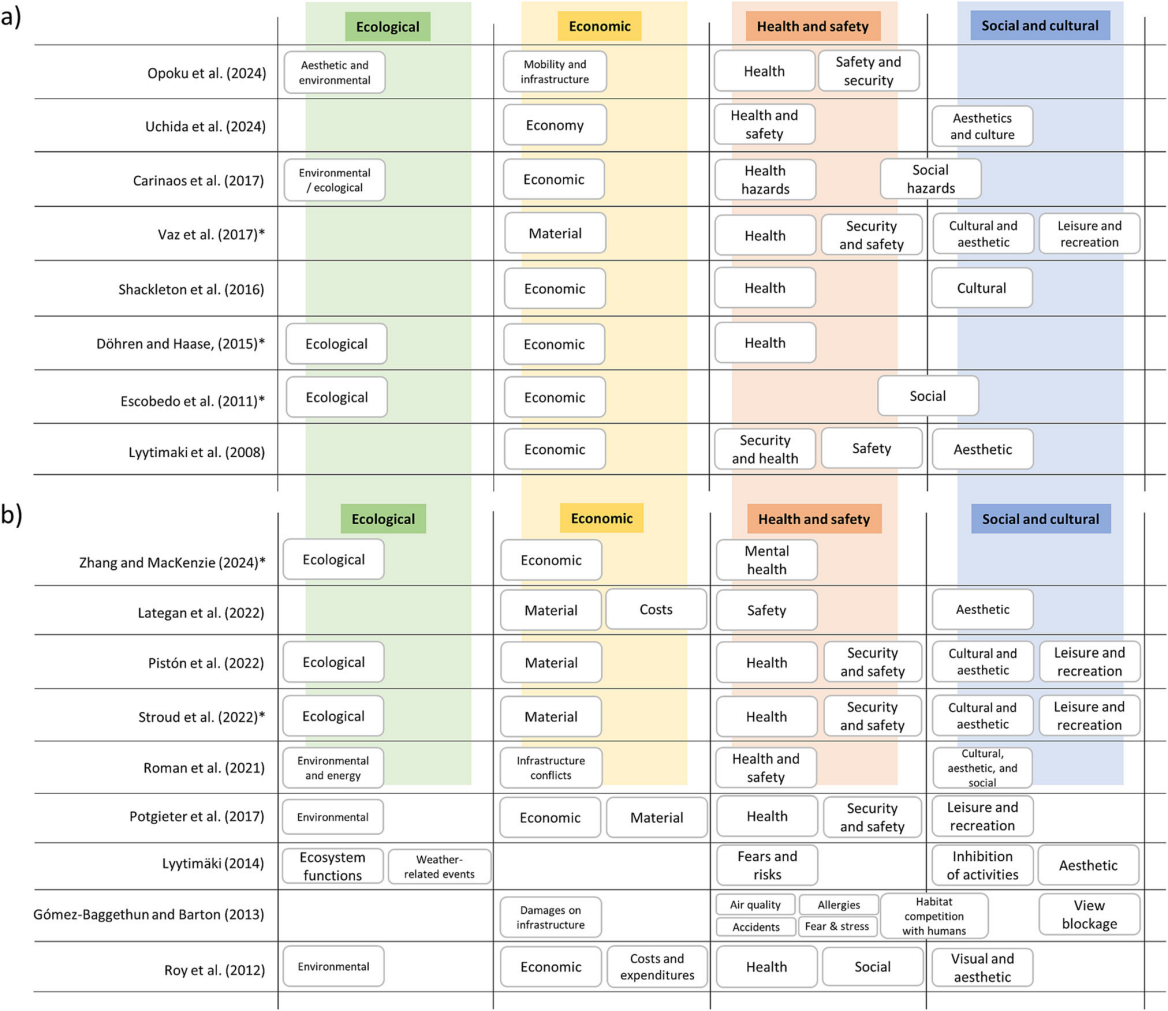
Tabular representation of how ecosystem disservice (ED) classifications from a) articles that propose a classification and b) articles that only apply a classification fit into 4 overarching ED boxes—ecological, economic, health and safety, and social and cultural. Note in part a) Guo et al. (2022) and Lliso et al. (2022); Soga and Gaston (2022); Ceauşu et al. (2019) and in part b) Andrade et al. (2024); Semeraro et al. (2021) are not shown given their high divergence in thematic classes (see Table 2B)

However, there is mostly strong overlap between the different classifications, with boxes representing the main spheres of ecological, economic, health and safety, and social and cultural (Fig. 3). Three articles do not include an ecological class (Gómez-Baggethun and Barton 2013; Shackleton et al. 2016; Vaz et al. 2017), while all classification systems include a health and safety class, all except Lyytimäki (2014) an economic class type, and all except Döhren and Haase (2015) and Opoku et al. (2024) a social and cultural class. We do not directly compare several of the classifications, including Semeraro et al. (2021); Guo et al. (2022) and Lliso et al. (2022) in Fig. 3, since their classes do not at all align. For example, Guo et al. (2022) use 6 ‘ecological context’ classes that include ‘urban forest’ and ‘plant invasions’; Lliso et al. (2022) use instrumental, intrinsic, and relational disvalues; and Semeraro et al. (2021) modify classes from Gómez-Baggethun and Barton (2013) and Döhren and Haase (2015) by using ‘ecosystem functions.’

Sometimes the same disservices are assigned to several different classifications by the different authors. For example, irritating sounds, smells, or behavior of organisms are assigned to aesthetic and cultural (Shackleton et al. 2016) and leisure and recreation (Vaz et al. 2017). Another example is allergic reactions to pollen, attributed to social nuisances (Escobedo et al. 2011), health (Vaz et al. 2017), externality-related costs (Vogt et al. 2015), and environmental/ecological (Cariñanos et al. 2017). In addition to the main classifications (Fig. 3), several authors also provide a second level of aggregation. Lategan et al. (2022) and Shackleton et al. (2016) also use higher-order ‘abiotic’ and ‘biotic’ groupings, Gómez-Baggethun and Barton (2013) use higher-order classes of ecosystem functions (e.g., photosynthesis, tree growth, aging of vegetation), while Lyytimäki (2014) further subdivide into ecosystem function or property.

Several prominent articles were not suitable for direct comparison among classes, but nevertheless present contributions to the topic. For example, Lyytimäki and Sipilä (2009) provide a useful breakdown of categories of driving forces of ED but otherwise nearly directly replicate the classification from Lyytimäki et al. (2008). Similarly, Lyytimäki (2017) adds ‘environmental and energy issues’ to a previous classification, but the classes provided are stated as examples rather than ‘a comprehensive overall picture.’ Mao and Cui (2021) use existing classes from Cariñanos et al. (2017) but do contribute a novel binary meta-classification of ‘actual perceived EDs’ and ‘potential EDs.’ The latter includes the fear and security of dark spaces and potential danger of aging tree species, among others. Finally, Wu et al. (2021) use MEA ES classes (MEA 2005) but also create the meta-classes of final (direct) and intermediate (indirect) bifurcation. In the former are impacts such as diseases or injuries, air or water quality impacts, and unpleasant feelings, while intermediate ED act to ‘increase the delivery of final ED…’ and include the introduction of invasive species and a decrease in soil nutrients.

### Ecosystem disservice frameworks

We found 23 articles that describe or apply a new or adapted framework. Of these, 8 articles propose context-specific frameworks including the analysis of fear of crime (Sreetheran and van den Bosch 2014), urban tree air quality (Eisenman et al. 2019), or invasive plants (Milanović et al. 2020). In this section, we focus on the 15 articles identified that propose frameworks dealing explicitly with ED as the main focus (Table 3).

**Table 3:** List of 15 articles from the review that propose frameworks dealing explicitly with ED as the main focus. Citation count, topic, and defining features of the frameworks are described

Article	Citations*	Framework purpose/topic	Defining features
Legg and Kabisch (2024)	4	Depict relationship between green space, pollen, and users’ perceptions, behaviors and mental health or well-being	Green space affects mental health but is mediated by individual characteristics and social context, including ED as environmental stressors (along with ES)
Opoku et al. (2024)	2	ES and ED classes and examples come together to affect human health and well-being in the context of urban forests and trees	Adapted from 11 different key articles; mostly a classification system that acknowledges mutual influence of ES and ED visually
Portoghesi et al. (2023)	6	Assessment referring to the ED caused by the fall of urban trees	Designed for the assessment of ED caused by the fall of urban trees; includes factors that affect ED and impacts on human well-being
Pereira et al. (2023)	34	Illustrate how NBS can lead to both ES and ED	Shows the impact on human well-being through nature-based solutions; includes ES and ED
Wu et al. 2021	31	Conceptual relationships between ecosystem structure, functions, disservices, negative effects and value loss	Cascade framework based on the work from Haines-Young and Potschin (2012) but includes ED instead of ES
Mao and Cui (2021)	0	ES and ED are directly correlated and arise from processes and functions of ecosystems	Simplified to show that ecosystem processes and functions can cause ES and ED with impacts on socio-economic system
Saunders (2020)	28	ES and ED are conceptualized as positive or negative outcomes of ecological interactions and processes	Decision tree for assessing the impact of ED with different criteria
Leong et al. (2020)	21	Relationship between socio-demographic factors, participation in nature-related activities, personal characteristics and the perception of ecosystem services and disservices	Shows relation between different socio-demographic and personal factors and the perception of ED and ES
Ceauşu et al. (2019)	111	Human–wildlife coexistence management, conceptualized in the ‘social-ecological framework for ecosystem disservices and services’ (SEEDS)	Includes subsystems of ES, ED, governance, wildlife units, and dis(service) recipients that lead to and receive feedback from different interactions and outcomes
Döhren and Haase (2019)	41	Flowchart of the assessment of various ED	Designed for the assessment of ED caused by the fall of street trees; includes different evaluation criteria
Barnaud et al. (2018)	187	Framework of analysis of social interdependencies underlying ecosystem services and disservice dynamics	Links providers, ES and ED, intermediaries, and beneficiaries. Cognitive framings, levels of organization, power relations, and institutions are acknowledged in contexts of the different actors
Campagne et al. (2018)	77	Distinguish between the processes or functions that result in ED	Shows the distinction between ED resulting from ecosystem management, ecological processes and as negative provision; includes the socio-economic system
Vaz et al. (2017)	253	Address both ED and ES in context of value attribution and social-ecological management	Addressing both ED and ES considering three different dimensions—ecological, social, and social ecological
Lyytimäki (2017)	66	Conceptual framework focusing on identification of ED AND ES and their management	Modified from Escobedo et al. (2011); includes ES and ED as end products of different interactions in urban ecosystems
Shackleton et al. (2016)	390	Show ED within the continuum between natural and social hazards	ED are shown within the continuum from natural to social hazards; integrated into the social-ecological system

^*^Citation numbers using Google Scholar—10 December 2024

The starting points of causal connections within frameworks include those with a strong direct link to ecosystem structures and processes (Shackleton et al. 2016; Vaz et al. 2017; Wu et al. 2021), and those with anthropocentric origins, including the differences in perception in relation to ES/ED (Leong et al. 2020). All of the cross-context frameworks use boxes and arrows to depict elements and flows except for Lyytimäki (2017), who use a Venn-diagram approach with overlapping ovals, and Shackleton et al. (2016), who only use one large arrow to represent a spectrum of ‘natural/geophysical hazards’ to ‘social hazards.’ In most cases, ED are seen as a direct result of biological processes (n = 11), the exceptions being the frameworks by Portoghesi et al. (2023), where disservices are caused by disturbances to the socio-biological system, Leong et al. (2020), where ED result from social differences, and Opoku et al. (2024), who only have ED and ES coming together to influence ‘human health and well-being.’

Structural variation in the frameworks is accompanied by differences in their visual origins (causal starting points). Most of the frameworks refer to terms with a biological background, such as ecological interactions and processes (Saunders 2020), biophysical structures or processes (Wu et al. 2021), or disturbance to the socio-biological urban system (Portoghesi et al. 2023) that lead to ED. This contrasts with the framework of Pereira et al. (2023), who focus on nature-based solutions as triggers for ED or ES. Shackleton et al. (2016) do not show a clear starting point, placing ED in the center of a continuum that grows out of the social-ecological system and can lead to natural or social hazards. Finally, Döhren and Haase (2019) integrate a classification of ED into a flow diagram that ends, rather than starts, with urban ED for assessment purposes.

In addition, frameworks vary based on their representation of a link between ED and ES. Shackleton et al. (2016), Döhren and Haase (2019), and Portoghesi et al. (2023) do not include ES. The same is true for Wu et al. (2021), who break ED down into boxes of intermediate and final ED. Their framework is structured in the same way as the ‘cascade model’ of ES (Haines–Young and Potschin 2012), thereby replicating the processes involved in the creation of ED as well as feedback and management (i.e., ED can either be mitigated or exacerbated by human action). In total, seven of the frameworks show that management can influence the ultimate impact of ED. Frameworks that do include ES (n = 11) link it to ED as potential bifurcated outcomes or as starting points of causal processes (Gutierrez–Arellano and Mulligan 2018; Opoku et al. 2024) or with ES and ED boxed together (e.g., ‘ecosystem (dis)services’) (e.g., Barnaud et al. 2018; Ceauşu et al. 2019; Legg and Kabisch 2024). However, there are differences in conceptualizing this connection, including linking ES and ED at the social-ecological interface (Vaz et al. 2017) or through the varying outcomes of the respective perception of individuals (Leong et al. 2020).

Including Leong et al. (2020), who have personal factors such as awareness, knowledge and nature relatedness at the center of their framework that determine final perception (similar to (Legg and Kabisch 2024)), six frameworks depict the individual assessment of ED in their structure. Several deal explicitly with different stakeholders and spaces (Campagne et al. 2018; Barnaud et al. 2018; Mao and Cui 2021), while others use a causal path diagram where different perceptions are differentiated and final disservices or impacts are determined as a result (Saunders 2020; Blanco et al. 2022; Legg and Kabisch 2024). In line with the individual perspective, Pereira et al. (2023) differentiate between positive/negative and actual/perceived impacts on human well-being, while Blanco et al. (2022) depict ‘mental models’ that create a feedback on ecosystems through management and coping practices.

Other forms of assessment include Döhren and Haase (2019) and Portoghesi et al. (2023), who show a scheme for the assessment of ED caused by urban trees and in particular by tree falls. The assessment by Döhren and Haase (2019) is represented as a flowchart, where various ED categories are shown at the beginning and the individual assessments of hazards and risks are indicated as a result at the final stage. The flowchart is influenced by various indicators and the social-technological context in the form of vulnerabilities, whereas Portoghesi et al. (2023) point out the different factors that have an influence on valuation (for example, by differentiating between current or perceived negative influences).

### Composite Ecosystem Disservices (CED) framework

**Fig. 4:**
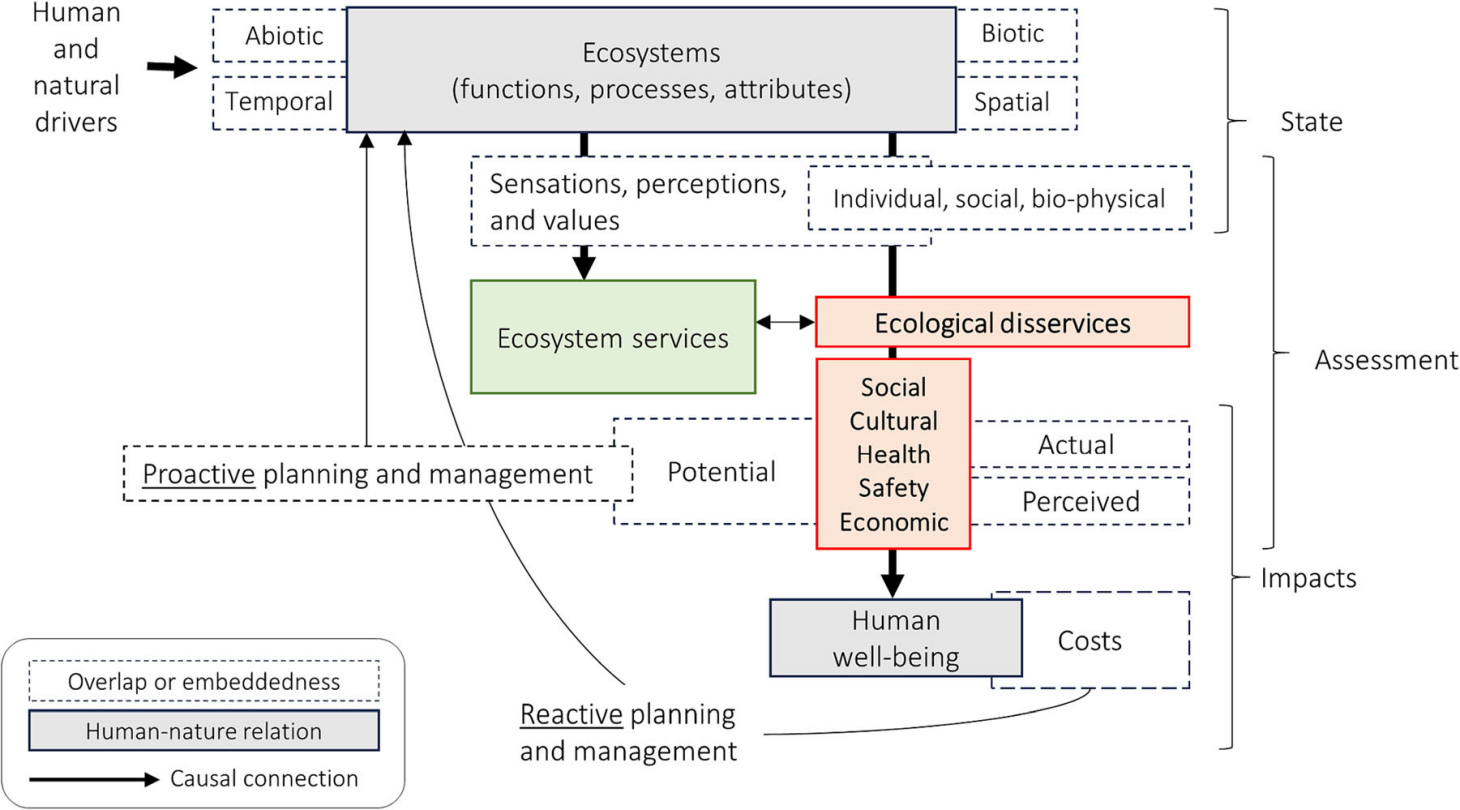
The Composite Ecosystem Disservices (CED) framework synthesizes knowledge from the reviewed frameworks and classifications, aiming to increase applicability across contexts and orient use toward research and policy for the management and mitigation of ED

Our Composite ED (CED) framework is created to highlight those aspects from existing frameworks that allow best for standardization and replication while not being too generic to lose impact. It is divided into three main sections that reflect key foci of the reviewed ED frameworks—state, assessment, and impacts. It follows a top to bottom, causal flow direction to align it with the popular ‘cascade model’ originally for ES (Groot et al. 2010; Haines-Young and Potschin 2012) and modified to include ED (Vogt et al. 2015; Campagne et al. 2018; Wu et al. 2021) (Fig. 4).

The **‘state**’ section of the CED takes as a starting point ecosystem functions, processes, and attributes that are influenced externally by the broad category of ‘human and natural drivers’ (e.g., land-use change, climate change, socio-natural disasters, invasive species, conservation efforts, etc.). The grey ecosystem box is the basis for phenomena that can be interpreted as ES or ED. We explicitly recognize the abiotic and biotic distinction among their elements (Shackleton et al. 2016; Vaz et al. 2017) and temporal and spatial variation within ecosystems that can lead to ED (Campagne et al. 2018). For example, disservices caused by tree fall can be short-term (within a few seconds) and widespread—initiated by an external driver such as a major storm (Conway and Yip 2016). In contrast, a disservice like damage to sidewalks is usually a highly localized and long-term process caused by natural root growth (Drillet et al. 2020).

The prominent green ES box represents the general abundance of benefits from ecosystems relative to ED. ES can often be enjoyed by a wider swath of people than are negatively affected by ED from the same ecosystem process or element (e.g., trees that lead to the individual experience of thermal comfort versus allergic reaction from pollen).

We use ‘ecological disservices’ as a root source of ED that generally only indirectly affect human well-being through more direct ED (social, cultural, health, safety, or economic) (Vaz et al. 2017). Ecological disservices thus include invasive species that affect the ecosystem (Lyytimäki 2017) or the emergence of pests that can cause lasting damage to trees or parks (Saunders and Luck 2016). Since they interact with the environment by impairing other ecological functions or displacing native species, the effects on humans are less direct (Shackleton et al. 2016; Roman et al. 2021) (e.g., ‘intermediate’ and ‘final’ ED according to Wu et al. (2021)). ES and ED are also related since a reduction of ES can equate to ED, while increasing ED can detract from ES (visually represented also by Campagne et al. (2018) and Mao and Cui (2021)). However, following Shackleton et al. (2016), ED must result from ecosystems themselves and thus all causes of reduced ES do not lead to increased ED.

In the ‘**assessment of impact’** section, we emphasize the role of diversity of people and place on the ultimate consideration of well-being and allow space for informal/individual ‘assessments’ as well as research or practice-oriented assessments. ‘Assessment’ overlaps with ‘state’ since the existence of ES and ED is dependent on the human perspective, while the ED themselves can also be defined as ‘actual’ or ‘perceived’ (Shackleton et al. 2016). In the CED, individual diversity goes beyond ‘sensations’ to include perceptions and values as well as individual and social attributes along with social diversity (Legg and Kabisch 2024) such as different interested stakeholders (Barnaud et al. 2018). For example, the importance of trees was shown to be valued differently by educated and less educated people (Fernandes et al. 2019), and women have been found more likely to perceive security risks in green spaces than men (Sreetheran and van den Bosch 2014). Additionally, the functions of trees can be perceived as positive or negative by people of different ages (Wolf et al. 2020). The ‘social attributes’ category integrates findings from Lyytimäki et al. (2008) and Lo and Jim (2010) in which perception was influenced by a park’s social setting in relation to different groups, such as criminal enterprises or gangs. In terms of actual frameworks, Sreetheran and van den Bosch (2014) visualize the perception of urban green spaces regarding the ED of fear; Lyytimäki (2017) uses nested overlapping classes of distinct ecological, social, and individual spheres that can impact health; and Hwang and Roscoe (2017) represent ‘site attributes’ and ‘socio-demographics’ as both influencing ‘perceived site attributes’ (which includes disservices).

The ‘actual’ and ‘perceived’ distinction follows the highly influential Shackleton et al. (2016), in which ED are defined as ‘perceived or actual negative impacts.’ We align with the perspective that both are equally ‘valid’ and important subjects of research and management, since, for example, only the fear of negative impacts can be just as strong (or stronger) a motivator for behavior (including ecosystem management). Biophysical attributes refer to, for example, the density of vegetation being perceived differently (Lyytimäki et al. 2008), or overgrown green spaces being identified as particularly ugly (Camacho-Cervantes et al. 2014). Finally, a temporal element differentiates potential impacts with actual or perceived ED since assessments can identify ED that may occur in the future (Mao and Cui 2021) and can be mitigated through proactive management (Shackleton et al. 2016).

We use the 5 most common direct ED classes identified in our review of social, cultural, health, safety, and economic. We view ‘material’ as highly related to economic, and thus do not explicitly include it (see ‘material’ in Potgieter et al. (2017) and Stroud et al. (2022)). The category of ‘cultural’ encompasses classes separated in past work such as ‘aesthetic’ (Lyytimäki 2014) or ‘leisure and recreation’ (Potgieter et al. 2017). This helps bring the ED classification and framework more in line with the commonly used MEA for ES (MEA 2005). Although we rely primarily on high-level aggregated classes, we also decided to separate ‘health’ and ‘safety.’ This recognizes the extensive literature on the allergens created by ecosystem elements, as well as issues such as mosquitos (Vaz et al. 2017; Mao and Cui 2021; Legg and Kabisch 2024). Safety is important because it goes beyond health to more strongly recognize subjective well-being—i.e., whether one ‘feels’ safe or not (Sreetheran and van den Bosch 2014), in line with the importance of individual and social perceptions. We exclude natural hazards as an explicit ED, which was included in, e.g., Lyytimäki (2014) and Shackleton et al. (2016), and rather assign these to the role of external drivers. For theoretical and methodological consistency, ED should be classified based on impact rather than cause and originate from ecosystem processes (Shackleton et al. 2016).

In the third and final ‘**impacts’** section, ED influence human well-being, which creates costs for individuals and society and, at this stage, should lead to reactive planning and management. Costs are considered separately from ED, as different costs may be incurred for individual stakeholders through a range of disservices (Barnaud et al. 2018; Blanco et al. 2020; Roman et al. 2021). For example, Escobedo et al. (2011) and Vogt et al. (2015) consider the various costs associated with urban forests, with the latter attributing ‘negative externalities’ to ED. Health costs include treating injuries or allergies originating from vegetation or animals (Escobedo et al. 2011), or fallen trunks or branches (Roman et al. 2021). Costs can lead to reactive management of green spaces (Gerstenberg and Hofmann 2016) through activities like planting, irrigation, the cutting of excessive growth (Vogt et al. 2015), repairing infrastructure from damage caused by roots (Lyytimäki et al. 2008; Drew-Smythe et al. 2023), or treatment of pests or diseases (Cariñanos et al. 2017). Finally, any reactive planning and management stemming from incurred costs should ideally feed into more proactive future planning and managing of ecosystems to minimize ED.

## Discussion

### Key findings and contributions

Despite the targeted uptake of several highly influential ED articles (e.g., Lyytimäki and Sipilä 2009; Döhren and Haase 2015; Shackleton et al. 2016), there is still high inconsistency among ED classifications. While this is understandable given that some frameworks are more useful when tailored to specific contexts (e.g., plant invasion (Vaz et al. 2017)), divergence among broad class names is not conducive to study comparison, replication, understanding, or much needed transdisciplinary research. The lack of consistently applied classification systems for ED research (including its neighboring concepts) supports similar findings from Guo et al. (2022). Unlike the concept of ES, in which several prominent classifications have emerged as ‘standards,’ research within the ED field either groups all ED together (especially if the primary focus of the paper is rather ES, e.g., Gómez-Baggethun and Barton 2013; Buij et al. 2017); picks and chooses classes and combines them from influential papers (e.g., Potgieter et al. 2017; Lategan et al. 2022); or creates tailor-made classification systems for specific contexts (e.g., Semeraro et al. 2021; Rodgman et al. 2024). This discrepancy in classification complicates the direct comparison of results (Delshammar et al. 2015). Animal excrement, for instance, can result in maintenance costs (Lyytimäki et al. 2008), but is also perceived as an unattractive nuisance, representing an aesthetic or social disservice for other authors (Shackleton et al. 2016; Mao and Cui 2021).This is not to imply that having different conceptualizations and classifications of ED (and ES) is inherently a drawback, nor that a single unified framework is wholly necessary. The plurality of frameworks is both inevitable and beneficial given the diverse scientific communities and disciplines engaged in this relatively young field of research. Still, referring to common framework elements and classes could ease the communication and facilitate a shared understanding of ED concepts for greater uptake in research and policy.

In this context, we identified and analyzed different ED classification systems in detail and ultimately proposed an overarching classification that can be used as a crosswalk between different classification systems. We identified 23 articles that include a new or adapted classification that is generalizable across contexts. Of these, 12 articles explicitly propose an ED classification, and 11 articles apply a classification (without explicitly encouraging its uptake) (Table 2). There is a high degree of overlap between the different classifications, with classes representing the main spheres of ecological, economic, health and safety, and social and cultural issues (Fig. 3). Four articles do not include an ecological class (Gómez-Baggethun and Barton 2013; Shackleton et al. 2016; Vaz et al. 2017; Uchida et al. 2024); all classification systems include a health and safety class type (included as ‘social’ in Escobedo et al. (2011)); all except Lyytimäki (2014) an economic class type; and all except Döhren and Haase (2015) and Opoku et al. (2024) a social and cultural class. Several articles diverge strongly despite being applicable across contexts, including Semeraro et al. (2021); Guo et al. (2022) and Lliso et al. (2022), which were therefore not directly comparable with the overarching categories. Ultimately, the classification presented in the CED attempts to bundle all relevant classes and terms so that future applications can follow a standardized approach of assigning various ED to a fixed class (Fig. 4). This contributes to the creation of a common classification that will support further research and facilitate consensus on ED and their study and management (Shackleton et al. 2016; Blanco et al. 2019).

The reviewed frameworks mostly over-simplify or omit key processes in the generation, assessment, or management of ED (e.g., Shackleton et al. 2016). Although we expect variation, many do not incorporate the important individual and sector-specific advancements made in ED research. Our work highlights similarities and unique attributes of frameworks, finding generally that many existing frameworks fail to integrate key elements of social-ecological systems that have implications for how ED are understood to be generated, assessed, and managed. This is in part due to different objectives regarding practicality and communication and the different disciplines involved in ED research. Nevertheless, frameworks that comprise causal connections, including with ES, allow for understanding how and when assessment, management, and mitigative action can occur to prevent or minimize ED.

The CED framework we present can support such consideration of systemic causes behind ED when applied within specific social-ecological contexts, along with providing a useful tool for visualization and communication in research and ultimately decision-making. For example, focusing on ED only as end-state impacts may neglect consideration of how actions such as the introduction and outbreak of harmful pests as a socio-natural driver of ecosystem change can contribute to the long-term development of ED (Lyytimäki 2017), as well as socio-natural hazards such as floods, fires, and severe storm events (Lyytimäki and Sipilä 2009; Conway and Yip 2016; Roman et al. 2021). Purely anthropogenic drivers and how they lead to ED should also be understood and can be dissected starting from the CED framework, including the general mismanagement of urban vegetation (Eisenman et al. 2019), landscape and species management (Ooba and Hayashi 2017; Pataki et al. 2021), and the neglect of spaces (Lyytimäki et al. 2008). Such mismanagement highlights the idea that while ES and ED occur depending on subjectivity and individual characteristics, steps must be taken to improve planning regardless. For example, the practice of planting exotic species because they are aesthetically pleasing is sometimes still given more weight than their potential underlying ecological disservices. In the CED framework, there is an interaction between the drivers, the ecosystem, and the ecological disservices, resulting ultimately in more direct ED that we present as five classes (i.e., social, cultural, health, safety, and economic). The CED framework classes draw from Fig. 3, which itself can be used as a ‘crosswalk’ between different classification systems. In this way, the CED framework and classes are a step toward a common valuation framework to better assess the direct impacts and consequences for human well-being and to support relevant stakeholders in reactive decision management and proactive planning stages (Lyytimäki 2015; Shackleton et al. 2016; Guo et al. 2022). Accordingly, the following questions regarding ED can be drawn by referring to the CED framework and should be clarified during the planning process or management: Where can ED occur? What causes them? Can they be prevented? How can they be prevented? What are the potential costs? and What trade-offs exist with ES?

### Limitations and future outlook

Our in-depth review is limited to articles that present a new or adapted ED classification or framework. The synthesized knowledge and lessons learned are presented in the CED framework. Further research should test the CED framework by applying past empirical ED articles to it, integrating it into ES and ED assessments and ongoing relevant research, and through further expert validation. Our classification system integrates lessons across relevant research fields in an effort toward standardization and replication of studies. However, to increase the utility of the classification system within ED assessments, more detailed sub-classes should be created in the form of a ‘look-up’ system comparable to the MEA or especially the CICES (Shackleton et al. 2016; Blanco et al. 2019). At the same time, it is crucial to recognize the importance of flexibility to account for the diverse social-ecological specificities within ED research. In this regard, we recognize that the IPBES approach of shifting from ES to defining relatively broad categories of ‘nature’s contributions to people’ (NCP) may have a corresponding role to play for the ED concept with ‘disvalues’ (IPBES 2022; Lliso et al. 2022). It is worth further research to explore whether the starting point of diverse values and conceptualizations of nature would allow for improved ED communication across worldviews and classification systems. As the field continues to grow, and methods that require specificity such as economic assessments of ED occur concurrently with ES, such detailed classification systems will be tested and become increasingly useful.

The ED literature overall is relatively limited, as evidenced by both the automated bibliometric and manual systematic reviews. This is particularly true in comparison with studies on ES, given the 535 articles explicitly using the term ED that we found compared to the over 50,000 explicitly using ES.^4^ A greater standardization and uptake of the ED concept may depend simply on more time to mature (the ES concept has its origins in the 1970s versus the 2000s for ED), or its promotion by similar international policy initiatives to those that carried ES forward such as the MEA, TEEB, and IPBES (Nápoles-Vértiz and Caro-Borrero 2024). The increasing prominence around trade-offs of poor planning or management in highly relevant fields such as climate change adaptation—e.g., ‘maladaptation,’ ‘response risks,’ or ‘risk transfer’ (IPCC 2022)—may also provide inroads for the consideration of ED.

In our review and subsequent development of the CED framework, we hypothesized that including a range of synonyms for ED would allow us to expand beyond any existing ED knowledge silo to include other relevant concepts (see Table 1 with key words). We identified only 5 articles that do not use the ED term explicitly but include a new class or framework. The results of our bibliometric analysis support the idea that many thematic clusters are relevant to the concept of ED (Fig. 1), much like the transdisciplinarity of the ES concept. For example, the concept of tree risk assessment appeared as a tangential research area, in which focus is on the potential hazards and costs of urban trees (Koeser et al. 2016; Klein et al. 2019). However, Roman et al. (2021) demonstrate the potential for further integration, as they explicitly embedded their research on tree risk assessment within the ED umbrella. Another example is public health research. A review by Wolf et al. (2020) aligns well with the ED concept since they identify the potential negative impacts of urban trees on human health, including allergies and fear of crime, but without explicitly using ED. This article was not included in our dataset given the lack of classification or framework. Such findings suggest the high growth potential and utility of the ED concept within relevant fields. They also show that our search for non-explicit ED research could be expanded to empirical articles that do not necessarily present new or adapted classification systems or frameworks. This would allow for a more in-depth review across different related fields and promote the integration of such studies under the ED umbrella. For example, the highly relevant topic of environmental justice within public health and planning research, in which ‘environmental benefits and burdens’ are discussed (Plieninger et al. 2013; Kronenberg et al. 2020), can link to ED and thereby promote research and ultimately policy across these currently disparate fields.

It is also recommended that non-English literature be included in order to acknowledge the diverse values and disvalues (or ED) of nature (IPBES 2022). This could create a more complete picture of the benefits and burdens of ecosystems and may encourage the concurrent consideration of ED along with standard ES assessments. We view this as a rather obvious contribution and agree with the discussion of Guo et al. (2022), where they imply that promotion and further understanding of ES-ED interlinkages is perhaps the most crucial path forward for increasing the uptake of ED research and its relevance for green management and planning and the design and implementation of nature-based solutions (Albert et al. 2020; Pereira et al. 2023).

The CED framework can help communicate findings and act as a tool for ED research and planning. The ‘direct’ ED within the CED framework (social, cultural, health, safety, and economic) represent a classification system that draws on the most widely used past classes. By building on existing research and integrating from the wide variety of themes relevant to ED, our classification and framework aims to promote transdisciplinary research on ED, support communication and replication across fields, and ultimately align such processes with the planning and management of ecosystems.

## Supplementary Information

Below is the link to the electronic supplementary material.

Supplementary file 1 (PDF 689 KB)

## Footnotes

1 See a recent review article by Nápoles-Vértiz and Caro-Borrero 2024 for a comprehensive list of ES definitions.

2 The phrase ‘box of rain’ here represents the duality of concepts and classes such as rain—being both a potential ES or ED depending on context, values, and perception. It originates as a metaphor for the planet earth from a song by the same name (composed by Phil Lesh and Robert Hunter of the Grateful Dead).

3 We use the Bibliometrix Tool in R (v. 4.3.3) (Aria and Cuccurullo 2017).

4 Web of Science search using ‘ecosystem service*’ as a ‘Topic’ on August 5, 2024.
