# Supplementary material for: Boxes of rain: A systematic review on the classes and frameworks of ecosystem disservices
Source: Ambio. 2025 Mar 13;54(9):1431–49. doi: 10.1007/s13280-025-02157-1 (PMC12307269; doi:10.1007/s13280-025-02157-1)
Supplement: Supplementary file 1 — (PDF 689 KB) [file 13280_2025_2157_MOESM1_ESM.pdf]

Ambio

Supplementary Information

This supplementary information has not been peer reviewed

Boxes of rain: A systematic review on the classes and frameworks of ecosystem disservices

ANDERSON, Carl Cyrus[1*]; METZEMACHER, Andreas[2, 3]; ADEM ESMAIL, Blal[2]

[1] Leibniz Universität Hannover, Institute of Environmental Planning, Herrenhäuser Str. 2, 30419 Hannover, Germany

[2] Ruhr University Bochum, Institute of Geography, Universitätsstr. 150, 44805 Bochum, Germany

[3] Kreisstadt Bergheim, Urban Planning Department, Bethlehemer Str. 9 - 11, 50126 Bergheim, Germany

Figure S1. Three-Field Plot generated using bibliometrix (Aria and Cuccurullo 2017) showing the top 10 cited references, authors' key words, and journal sources (left to right) within the 448 articles in the larger ED dataset. One reference is in gray since it is not an article but rather refers to the (highly cited) statistical package R.

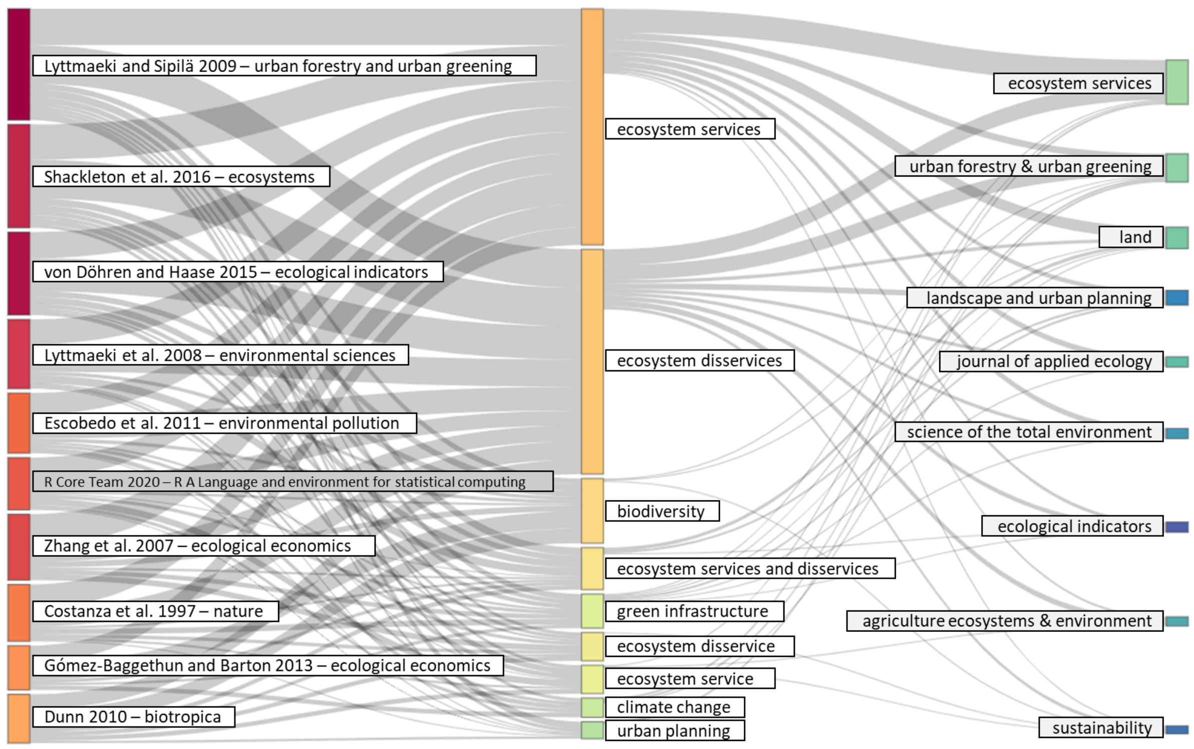

Text S1. List of the 48 reviewed papers that introduce a new or adapted ecosystem disservice classification or framework

1. Andrade, Mónica; Fernandes, Cláudia; Coutinho, António; Figueiredo, Albano (2024): Urban Green Infrastructure: Does Species' Origin Impair Ecosystem Services Provision? In Land 13 (1), p. 23. DOI: 10.3390/land13010023.

2. Barnaud, Cécile; Corbera, Esteve; Muradian, Roldan; Salliou, Nicolas; Sirami, Clélia; Vialatte, Aude et al. (2018): Ecosystem services, social interdependencies, and collective action: a conceptual framework. In Ecology and Society 23 (1), pp. 1–15.

3. Barnes, Michael R.; Nelson, Kristen C.; Dahmus, Maria E. (2020): What's in a yardscape? A case study of emergent ecosystem services and disservices within resident yardscape discourses in Minnesota. In *Urban Ecosyst* 23 (6), pp. 1167–1179. DOI: 10.1007/s11252-020-01005-2.

4. Blanco, Julien; Bellón, Beatriz; Barthelemy, Liane; Camus, Baptiste; Jaffre, Louise; Masson, Anne-Sophie et al. (2022): A novel ecosystem (dis)service cascade model to navigate sustainability problems and its application in a changing agricultural landscape in Brazil. In Sustain Sci 17 (1), pp. 105–119. DOI: 10.1007/s11625-021-01049-z.

5. Blanco, Julien; Sourdril, Anne; Deconchat, Marc; Barnaud, Cécile; San Cristobal, Magali; Andrieu, Emilie (2020): How farmers feel about trees: Perceptions of ecosystem services and disservices associated with rural forests in southwestern France. In Ecosystem Services 42, p. 101066. DOI: 10.1016/j.ecoser.2020.101066.

6. Broome, James David; Cook, David; Davíðsdóttir, Brynhildur (2024): Heavenly lights: An exploratory review of auroral ecosystem services and disservices. In Ecosystem Services 67, p. 101626. DOI: 10.1016/j.ecoser.2024.101626.

7. Campagne, Carole Sylvie; Roche, Philip K.; Salles, Jean-Michel (2018): Looking into Pandora's Box: Ecosystem disservices assessment and correlations with ecosystem services. In *Ecosystem Services* 30, pp. 126–136. DOI: 10.1016/j.ecoser.2018.02.005.

8. Cariñanos, Paloma; Calaza-Martínez, Pedro; O'Brien, Liz; Calfapietra, Carlo (2017): The Cost of Greening: Disservices of Urban Trees. In David Pearlmutter, Carlo Calfapietra, Roeland Samson, Liz O'Brien, Silvija Krajter Ostoić, Giovanni Sanesi, Rocío Del Alonso Amo (Eds.): The Urban Forest, vol. 7. Cham: Springer International Publishing (Future City), pp. 79–87.

9. Ceauşu, Silvia; Graves, Rose A.; Killion, Alexander K.; Svenning, Jens-Christian; Carter, Neil H. (2019): Governing trade-offs in ecosystem services and disservices to achieve human-wildlife coexistence. In Conservation biology : the journal of the Society for Conservation Biology 33 (3), pp. 543–553. DOI: 10.1111/cobi.13241.

10. Dobbs, Cynnamon; Kendal, Dave; Nitschke, Craig R. (2014): Multiple ecosystem services and disservices of the urban forest establishing their connections with landscape structure and sociodemographics. In *Ecological indicators* 43, pp. 44–55. DOI: 10.1016/j.ecolind.2014.02.007.

11. Döhren, Peer von; Haase, Dagmar (2015): Ecosystem disservices research: A review of the state of the art with a focus on cities. In *Ecological indicators* 52, pp. 490–497. DOI: 10.1016/j.ecolind.2014.12.027.

12. Döhren, Peer von; Haase, Dagmar (2019): Risk assessment concerning urban ecosystem disservices: The example of street trees in Berlin, Germany. In *Ecosystem Services* 40, p. 101031. DOI: 10.1016/j.ecoser.2019.101031.

13. Escobedo, Francisco J.; Kroeger, Timm; Wagner, John E. (2011): Urban forests and pollution mitigation: analyzing ecosystem services and disservices. In *Environmental pollution (Barking, Essex : 1987)* 159 (8-9), pp. 2078–2087. DOI: 10.1016/j.envpol.2011.01.010.

14. Gómez-Baggethun, Erik; Barton, David N. (2013): Classifying and valuing ecosystem services for urban planning. In *Ecological Economics* 86, pp. 235–245. DOI: 10.1016/j.ecolecon.2012.08.019.

15. Guo, Rong-Zhi; Song, Yao-Bin; Dong, Ming (2022): Progress and Prospects of Ecosystem Disservices: An Updated Literature Review. In *Sustainability* 14 (16), p. 10396. DOI: 10.3390/su141610396.

16. Gutierrez-Arellano, Claudia; Mulligan, Mark (2018): A review of regulation ecosystem services and disservices from faunal populations and potential impacts of agriculturalisation on their provision, globally. In NC 30, pp. 1–39. DOI: 10.3897/natureconservation.30.26989.

17. Lategan, Louis Gerhardus; Steynberg, Zene; Cilliers, Elizelle Juanee; Cilliers, Sarel Stephanus (2022): Economic Valuation of Urban Green Spaces across a Socioeconomic Gradient: A South African Case Study. In *Land* 11 (3), p. 413. DOI: 10.3390/land11030413.

18. Legg, Rupert; Kabisch, Nadja (2024): The effects of allergenic pollen in green space on mental health, behaviour and perceptions: A systematic review. In Urban Forestry & Urban Greening 92, p. 128204. DOI: 10.1016/j.ufug.2024.128204.

19. Leong, Rachel A.T.; Fung, Tze Kwan; Sachidhanandam, Uma; Drillet, Zuzana; Edwards, Peter J.; Richards, Daniel R. (2020): Use of structural equation modeling to explore influences on perceptions of ecosystem services and disservices attributed to birds in Singapore. In *Ecosystem Services* 46, p. 101211. DOI: 10.1016/j.ecoser.2020.101211.

20. Lliso, Bosco; Lenzi, Dominic; Muraca, Barbara; Chan, Kai M. A.; Pascual, Unai (2022): Nature's disvalues: what are they and why do they matter? In *Current Opinion in Environmental Sustainability* 56, p. 101173. DOI: 10.1016/j.cosust.2022.101173.

21. Lyytimäki, Jari (2014): Bad nature: Newspaper representations of ecosystem disservices. In *Urban Forestry & Urban Greening* 13 (3), pp. 418–424. DOI: 10.1016/j.ufug.2014.04.005.

22. Lyytimäki, Jari (2017): Disservices of urban trees. In Francesco Ferrini, Cecil C. Konijnendijk den van Bosch, Alessio Fini (Eds.): Routledge handbook of urban forestry. London, New York: Routledge/Taylor & Francis, pp. 164–176.

23. Lyytimäki, Jari; Petersen, Lars Kjerulf; Normander, Bo; Bezák, Peter (2008): Nature as a nuisance? Ecosystem services and disservices to urban lifestyle. In *Environmental Sciences* 5 (3), pp. 161–172. DOI: 10.1080/15693430802055524.

24. Mao, Liang; Cui, Xinyue (2021): Balanced Consideration of Urban Park Green Space to Provide Services and Disservices. In *Journal of Harbin Institute of Technology (New Series)* (28), Article 4, pp. 40–48.

25. Milanović, Marija; Knapp, Sonja; Pyšek, Petr; Kühn, Ingolf (2020): Linking traits of invasive plants with ecosystem services and disservices. In *Ecosystem Services* 42, p. 101072. DOI: 10.1016/j.ecoser.2020.101072.

26. Nicolás-Ruiz, Néstor; Suárez, María Luisa; Vidal-Abarca, María Rosario; Quintas-Soriano, Cristina (2024): Can dry rivers provide a good quality of life? Integrating beneficial and detrimental nature's contributions to people over time. In Ambio. DOI: 10.1007/s13280-024-02072-x.

27. Opoku, Patrick; Kwame Simpeh, Eric; Mensah, Henry; Akoto, Dorothy Asare; Weber, Norbert (2024): Perception of the services and disservices from urban forest and trees in the Garden City of West Africa. In Trees, Forests and People 16, p. 100550. DOI: 10.1016/j.tfp.2024.100550.

28. Pereira, Paulo; Yin, Caichun; Hua, Ting (2023): Nature-based solutions, ecosystem services, disservices, and impacts on well-being in urban environments. In *Current Opinion in Environmental Science & Health* 33, p. 100465. DOI: 10.1016/j.coesh.2023.100465.

29. Pistón, Nuria; Silva Filho, Dario S.E.; Dias, André T.C. (2022): Social inequality deeply affects people's perception of ecosystem services and disservices provided by street trees. In Ecosystem Services 58, p. 101480. DOI: 10.1016/j.ecoser.2022.101480.

30. Portoghesi, Luigi; Masini, Emanuela; Tomao, Antonio; Agrimi, Mariagrazia (2023): Could climate change and urban growth make Europeans regard urban trees as an additional source of danger? In *Front. For. Glob. Change* 6, Article 1155016. DOI: 10.3389/ffgc.2023.1155016.

31. Potgieter, Luke J.; Gaertner, Mirijam; Kueffer, Christoph; Larson, Brendon M. H.; Livingstone, Stuart W.; O'Farrell, Patrick J.; Richardson, David M. (2017): Alien plants as mediators of ecosystem services and disservices in urban systems: a global review. In *Biol Invasions* 19 (12), pp. 3571–3588. DOI: 10.1007/s10530-017-1589-8.

32. Rodgman, Mary Kathryn; Anguelovski, Isabelle; Pérez-del-Pulgar, Carmen; Shokry, Galia; Garcia-Lamarca, Melissa; Connolly, James J.T. et al. (2024): Perceived urban ecosystem services and disservices in gentrifying neighborhoods: Contrasting views between community members and state informants. In *Ecosystem Services* 65, p. 101571. DOI: 10.1016/j.ecoser.2023.101571.

33. Rodríguez-Morales, Beatriz; Roces-Díaz, José V.; Kelemen, Eszter; Pataki, György; Díaz-Varela, Emilio (2020): Perception of ecosystem services and disservices on a peri-urban communal forest: Are landowners' and visitors' perspectives dissimilar? In *Ecosystem Services* 43, p. 101089. DOI: 10.1016/j.ecoser.2020.101089.

34. Roman, Lara A.; Conway, Tenley M.; Eisenman, Theodore S.; Koeser, Andrew K.; Ordóñez Barona, Camilo; Locke, Dexter H. et al. (2021): Beyond 'trees are good': Disservices, management costs, and tradeoffs in urban forestry. In *Ambio* 50 (3), pp. 615–630. DOI: 10.1007/s13280-020-01396-8.

35. Roy, Sudipto; Byrne, Jason; Pickering, Catherine (2012): A systematic quantitative review of urban tree benefits, costs, and assessment methods across cities in different climatic zones. In *Urban Forestry & Urban Greening* 11 (4), pp. 351–363. DOI: 10.1016/j.ufug.2012.06.006.

36. Russo, Alessio; Escobedo, Francisco J.; Cirella, Giuseppe T.; Zerbe, Stefan (2017): Edible green infrastructure: An approach and review of provisioning ecosystem services and disservices in urban environments. In *Agriculture, Ecosystems & Environment* 242, pp. 53–66. DOI: 10.1016/j.agee.2017.03.026.

37. Saunders, Manu E. (2020): Conceptual ambiguity hinders measurement and management of ecosystem disservices. In *J Appl Ecol* 57 (9), pp. 1840–1846. DOI: 10.1111/1365-2664.13665.

38. Semeraro, Teodoro; Scarano, Aurelia; Buccolieri, Riccardo; Santino, Angelo; Aarrevaara, Eeva (2021): Planning of Urban Green Spaces: An Ecological Perspective on Human Benefits. In *Land* 10 (2), p. 105. DOI: 10.3390/land10020105.

39. Shackleton, C. M.; Ruwanza, S.; Sinasson Sanni, G. K.; Bennett, S.; Lacy, P. de; Modipa, R. et al. (2016): Unpacking Pandora's Box: Understanding and Categorising Ecosystem Disservices for Environmental Management and Human Wellbeing. In *Ecosystems* 19 (4), pp. 587–600. DOI: 10.1007/s10021-015-9952-z.

40. Sladonja, Barbara; Sušek, Marta; Guillermic, Julia (2015): Review on Invasive Tree of Heaven (Ailanthus altissima (Mill.) Swingle) Conflicting Values: Assessment of Its Ecosystem Services and Potential Biological Threat. In *Environmental management* 56 (4), pp. 1009–1034. DOI: 10.1007/s00267-015-0546-5.

41. Soga, Masashi; Gaston, Kevin J. (2022): The dark side of nature experience: Typology, dynamics and implications of negative sensory interactions with nature. In People and Nature 4 (5), pp. 1126–1140. DOI: 10.1002/pan3.10383.

42. Stroud, Sebastian; Peacock, Julie; Hassall, Christopher (2022): Vegetation-based ecosystem service delivery in urban landscapes: A systematic review. In *Basic and Applied Ecology* 61, pp. 82–101. DOI: 10.1016/j.baae.2022.02.007.

43. Uchida, Kenta; Blumstein, Daniel T.; Soga, Masashi (2024): Managing wildlife tolerance to humans for ecosystem goods and services. In Trends in ecology & evolution 39 (3), pp. 248–257. DOI: 10.1016/j.tree.2023.10.008.

44. Vaz, Ana S.; Kueffer, Christoph; Kull, Christian A.; Richardson, David M.; Vicente, Joana R.; Kühn, Ingolf et al. (2017): Integrating ecosystem services and disservices: insights from plant invasions. In *Ecosystem Services* 23, pp. 94–107. DOI: 10.1016/j.ecoser.2016.11.017.

45. Vaz, Ana S.; Kueffer, Christoph; Kull, Christian A.; Richardson, David M.; Vicente, Joana R.; Kühn, Ingolf et al. (2017): Integrating ecosystem services and disservices: insights from plant invasions. In *Ecosystem Services* 23, pp. 94–107. DOI: 10.1016/j.ecoser.2016.11.017.

46. Vogt, Jess; Hauer, Richard; Fischer, Burnell (2015): The Costs of Maintaining and Not Maintaining the Urban Forest: A Review of the Urban Forestry and Arboriculture Literature. In *AUF* 41 (6). DOI: 10.48044/jauf.2015.027.

47. Wu, Shuyao; Li, Binbin V.; Li, Shuangcheng (2021): Classifying ecosystem disservices and valuating their effects - a case study of Beijing, China. In *Ecological indicators* 129, p. 107977. DOI: 10.1016/j.ecolind.2021.107977.

48. Zhang, Baige; MacKenzie, Andrew (2024): Trade-offs and synergies in urban green infrastructure: A systematic review. In Urban Forestry & Urban Greening 94, p. 128262. DOI: 10.1016/j.ufug.2024.128262.

Table S1. 13 new or adapted *context-specific* classifications found in the literature review.

Article	Purpose / topic	Classes	Defining features
Barnes et al. 2020	Inductive coding of homeowner survey responses about lawn care	animals, plants, inputs, expectations	Expectations include pressure from neighbors to maintain the yard and potential embarrassment
Blanco et al. 2022	Synthesize insights from 'changing agricultural landscape in Brazil'	Material, indirect, health and security	Coded from farmer interviews
Blanco et al. 2020	Farmer perceptions of trees in rural forests in France	Material, agricultural-related, health/security	Coded from farmer interviews
Broome et al. 2024	Review of auroral ES and ED	Provisioning, regulation and maintenance, cultural; abiotic vs. biotic	Combines CICES with Wu et al. (2021) classes
Dobbs et al. 2014	Urban forests	allergen potential; damage to infrastructure potential	Highlights risk of trees at the end of their life expectancy
Gutierrez-Arellano and Mulligan 2018	Animal ES and ED and impacts of agriculturalisation on their provision	Invasive pollinators, herbivore insects, birds and mammals, invasive hosts, invasive frugivores and herbivores	Based on animal source
Rodgman et al. 2024	Perceived urban ED in gentrifying neighborhoods	economic, physical hazards, psychological, other social impacts	Argues the need to consider all ecosystem services and disservices to 'design green just cities.'
Nicolás-Ruiz et al., 2024	'Detrimental NCPs' in context of dry rivers with case study in south-eastern Spain	regulation of hazards and extreme events; regulation of detrimental organisms; regulation of freshwater quantity, location and timing; physical and psychological experiences	Uses NCP framing to discuss ED as 'detrimental NCP categories'

Vogt et al. 2015*	Costs of maintaining and not maintaining urban forests	Direct costs (of provisioning and maintaining trees); infrastructure interference costs; externality-related costs; opportunity costs	Opportunity costs are argued to be missing from urban forestry and arboriculture literature
Rodríguez-Morales et al. 2020	Landowner and visitor perceptions in a peri-urban communal forest	scariness; unpleasantness	Adds these two ED on a list of CICES v5.1 ecosystem services
Russo et al. 2017*	Different edible green infrastructure types	Edible urban forests and urban greening; school gardens; allotment gardens and community gardens; domestic gardens; historic gardens and parks and botanical gardens; green roofs and vegetable raingardens; edible green walls and facades	Classification based on geographic space of exposure
Sladonja et al. 2015	Negative effects of an invasive tree species in Europe	biodiversity decrease; invasive properties; human perspective	First two classes represent indirect conditions that can lead to actual impacts in the third class
Milanovic et al. 2020	Invasive plant species in Europe	18 unique classes corresponding to Latin species' names	Each species is associated with difference services and disservices
